# Employer Attractiveness From an Employee Perspective: A Systematic Literature Review

**DOI:** 10.3389/fpsyg.2022.858217

**Published:** 2022-06-01

**Authors:** Anke Dassler, Svetlana N. Khapova, Evgenia I. Lysova, Konstantin Korotov

**Affiliations:** ^1^Vrije Universiteit Amsterdam, Amsterdam, Netherlands; ^2^European School of Management and Technology Berlin, Berlin, Germany

**Keywords:** employer attractiveness, employer brand, employees, organizational commitment, organizational identification

## Abstract

With the growing interest in employer attractiveness, research is unsystematic on how this phenomenon can be conceptualized and studied. Studies tend to make little conceptual differentiation regarding for whom employers should be attractive, and therefore, address the perspectives of potential as well as current employees, who work in organizations for long periods of time. In this study our arguments relate to the phenomenon’s conceptual clarity as well as its differentiation from other related concepts. By focusing on employer attractiveness for current employees, we have systematically reviewed 48 studies published in business and management journals, and categorized findings into the Inputs–Mediators–Outputs model. This approach allowed us to depict significant limitations in the existing knowledge about employer attractiveness from the current employees’ perspective, and offer avenues for future research. Next, to delineate the future research agenda, we have suggested that employer branding in organisations needs to be targeted more toward current employees.

## Introduction

*“By* 2030, *we can expect a talent deficit of 85.2 million workers across the economies analyzed—greater than the current population of Germany. This global skills shortage could result in $8.452 trillion in unrealized annual revenue by 2030 – equivalent to the combined GDP of Germany and Japan.” (Korn Ferry Study, 2018)*

The literature on how to position organizations as attractive employers has been intensively developed during the last few decades since Ambler and Barrow (1996) published a study focusing on “employer branding.” While attracting the best candidates is still of high importance to organizations, it also becomes increasingly important for them to motivate their employees to stay on, and contribute to the organization for a long period (Tanwar and Prasad, 2017), mainly for two reasons. First, the demand for the acquisition of appropriate employees is rising, and organizations across different industries face challenges in finding the right people in the labor market. A recent study by Korn Ferry estimated that by 2030, there will be a talent deficit (meaning skilled employees) of 85.2 million workers across the different economies and industries analyzed (Korn Ferry Study, 2018).

Second, the possibilities of people sharing their experiences with organizations on social media channels like LinkedIn could lead to higher transparency, putting the spotlight on the reality, i.e., what employees actually face within organizations (Dabirian et al., 2017). Therefore, external employer branding without focusing on internally perceived attractiveness is not as effective as it was previously. Moreover, the boundaries of organizations are vanishing, leading to a world, wherein, the existing employees’ perceptions of organizational attractiveness influence companies’ brands in the context of different marketplaces (Mokina, 2014).

Yet, as is evident from recent studies and literature reviews, researchers continue to study employer attractiveness mainly by focusing on potential job applicants and future employees (Lievens and Slaughter, 2016; Theurer et al., 2018). Till date, little research has been conducted for understanding what shapes current employees’ perspectives relating to attractiveness of their employers.

This study aims to fill this gap. Since some evidence suggests that the attributes valued by employees are different from those valued by job seekers (Lievens, 2007; Reis et al., 2017), the aim of our systematic review is to contribute to employer attractiveness literature by providing a structured analysis on the basis of our knowledge on organizational actions that influence current employees’ perceptions of employer attractiveness, and their consequences. With this focus, we shall make several distinct contributions to the literature on employer attractiveness. First, this study synthesizes research evidence on internally perceived organizational attractiveness. In this way, it uniquely contributes to the existing literature reviews by summarizing concepts that were used to study employer attractiveness as perceived by current employees. Second, we shall cluster the findings of the different studies not only into antecedents and consequences of employer attractiveness, but also provide a structure for the different factors influencing internally perceived employer attractiveness. Third, this study discusses the practical implications for organizations regarding their employer branding activities and provides suggestions for future research.

## Research Methods

We drew on the *Web of Science (WoS)* database to search for relevant articles, using the general database. The results of an initial search using the term “employer attractiveness” indicated that it was too narrow since several highly cited scientific papers were missing, leading to a revision of the search strings to “employer brand” OR “employer perception” OR “employer value” OR “employer attractiveness” OR “employer reputation” OR “employer image” OR “employer branding” OR “organizational attractiveness”. While for the search within WoS no limitations were made, except for articles and reviews in English, we acknowledge that other bodies of research were not considered, especially books or other science publications.

**FIGURE 1:**
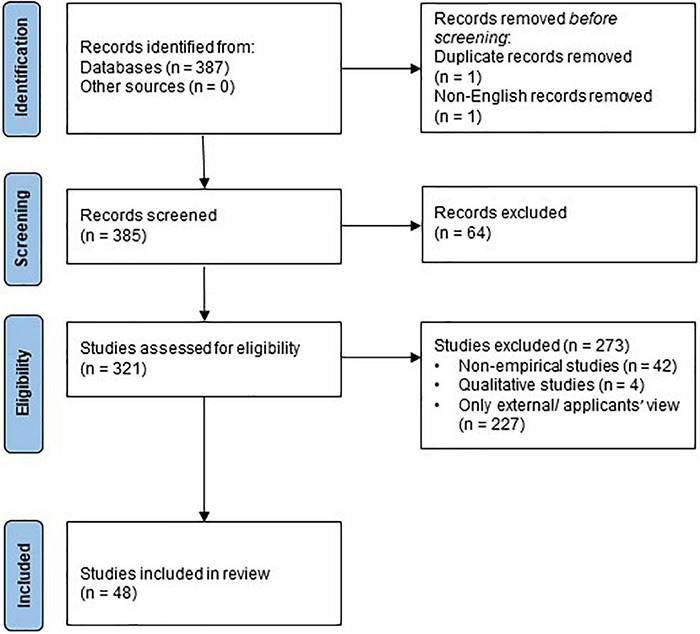
PRISMA diagram.

The review has been prepared according to PRISMA 2020 (Page et al., 2021). The search string initially resulted in a total number of 387 items, including empirical studies and reviews, and after the removal of a double entry and a non-English paper, 385 papers were reviewed. This led to the exclusion of an additional 64 papers, that did not focus on employer attractiveness in a broader sense, leaving 321 papers, of which 279 were empirical studies and 42 were reviews or conceptual papers. The 279 empirical studies were reviewed for a second time to distinguish whether the empirical work had been done with potential employees, such as job seekers (external view: 227 items), current employees (internal view: 46 items), or both (external and internal view: 6 items). The full text versions of the 52 empirical studies focusing on current employees, or both, potential and current employees were sourced, downloaded, and analyzed. A data extraction table was devised to record the evaluation, each item was read in full, and the data extraction table was then completed. Out of the studies, 48 were quantitative, which are listed in Tables 1,2. The four qualitative studies were considered together with the relevant conceptual papers and reviews. The study selection process is illustrated in Figure 1.

**TABLE 1:** Summary of quantitative empirical studies with employer attractiveness or related concepts as dependent variables (DV).

Nr.^a^	Author(s) (year)	Journal	Country	Dependent variables	Input factors and moderators
1	Dabirian et al. (2019)	IT Professional	Multiple (glassdoor)	Organisational attractiveness	*Antecedents*: different attributes of employer brand (economic value, development value, social value, work value and employer reputation)
2	Robertson et al. (2019)	Journal of Business-to-Business Marketing	Multiple (glassdoor)	Ranking of brand personalities	Different personality traits based on the brand personality dimensions; social media presence
3	De Waal (2018)	Journal of Organisational Effectiveness-People and Performance	Netherlands	Employer attractiveness	*Antecedents*: Happiness at work (HAW), High-performance organisation (HPO)
4	Maurya and Agarwal (2018)	International Journal of Organisational Analysis	India	Employer brand	*Antecedent*: Talent management, e.g., fair rewards and remunerates, work-life-balance, attraction and recruiting of talent
5	Vnouckova et al. (2018)	Engineering Economics	Czechia	Employer brand	*Antecedents*: talent management programs
6	Dabirian et al. (2017)	Business Horizons	Multiple	Employer attractiveness	Word-of-mouth and crowdsourcing
7	O’Neill et al. (2009)	Polish Journal Of Management Studies	India	Employer of choice	*Antecedents:* work culture (belongingness, fun work culture, opportunities for growth builds self-esteem, encourages creativity), company brand; stakeholder proposition, compensation and benefits, performance management, career anchors
8	Reis et al. (2017)	Personnel Review	Brazil	Employer attractiveness	*Antecedents*: workplace authenticity *Moderators*: gender, age, hierarchical level
9	Tanwar and Prasad (2017)	Personnel Review	India	Employer brand	*Antecedents*: healthy work atmosphere, training and development, work-life-balance, ethics, CSR, compensation and benefits
10	App and Buettgen (2016)	Employee Relations	Germany	Employer brand commitment	*Antecedents*: perceived organisational support (POS) and perceived supervisory support (PSS) *Mediators*: brand distinctiveness, brand prestige, brand trust
11	Biswas and Suar (2016)	Journal of Business Ethics	India	Employer brand(ing); attraction, retention; brand loyalty and employee engagement; performance	*Antecedents*: realistic job previews, perceived organisational support (POS), equity in reward administration, perceived organisational prestige, organisational trust, leadership of top management, psychological contract obligation, corporate social responsibility (CSR)
12	Fernandez-Lores et al. (2016)	BRQ-Business Research Quarterly	Unknown	Long-term orientation, enthusiasm with employer brand	*Antecedents*: Sensory, intellectual, emotional experience *Mediator*: affective commitment toward employer brand
13	Poulis and Wisker (2016)	Journal of Product and Brand Management	United Kingdom, United Arab Emirates	Employee-based-brand-equity, brand endorsement, brand allegiance, brand consistent behaviour	*Antecedents:* government and policy uncertainties, macroeconomic uncertainties, resources and services uncertainties, product and market demand uncertainties, competition uncertainties, technology uncertainties *Mediators:* perceived environmental uncertainty on a firm’s performance *Moderators*: people – average age, years in the firm, age as middle managers, organisations- years of operation, number of employees
14	Chawla and Lenka (2015)	Journal of Workplace Learning	India	Employer branding	*Antecedents*: intrapreneurship, knowledge management, total quality management *Mediator*: learning organisation
15	Sengupta et al. (2015)	Decision	India	Employer brand	*Antecedents:* Competitive pay and facilities, work-life-balance, challenging and interesting work, work environment – relationship with peers and supervisor, skills utilization, job
					security, recognition of potential, moral practices of managers, transparent company policies, training and development, kept promises, company brand, hierarchical position, contributing to organisational objectives, office infrastructure, duty hours, quick growth, stretched assignment, emotionally connecting with organisation and job, transferability of the job *Moderators*: age; gender; sector, hierarchical position
16	Trybou et al. (2014)	BMC Health Services Research	Belgium	Hospital attractiveness	*Antecedents*: economic attributes (pay and financial benefits, security), relational attributes (organisational support, leader support, work-life balance), professional attributes (hospital prestige, professional development opportunities)
17	Roongrerngsuke and Liefooghe (2013)	Asia Pacific Business Review	India, China, Thailand	Employer attractiveness	*Antecedents:* Competitive rewards, company’s brand and image, organisation’s stability and sustainability, job stability and security, job fit, job’s content/challenging job, good physical working conditions, position and status, supportive colleagues, learning and growth opportunities, career development and advancement, convenient location *Moderators: generation/age, Baby Boomer, cultural differences*
18^b^	Lievens (2007)	Human Resource Management	Belgium	Employer attractiveness	*Antecedents*: job or organisational characteristics (Social/team activities, physical activities, structure, advancement travel opportunities, pay and benefits, job security, educational opportunities, task diversity) and symbolic trait inferences (sincerity, excitement, cheerfulness, competence, prestige, ruggedness)

*^a^Studies are listed by the year of publication from latest to earliest, in alphabetical order.*

*^b^Study focused on both, potential and existing employees.*

**TABLE 2:** Summary of quantitative studies with employer attractiveness or related concepts as independent variables (IV) or a mediator.

Nr.^a^	Author(s) (year)	Journal	Country	Dependent variables	Input factors and moderators
1	Arasanmi and Krishna (2019)	Industrial and Commercial Training	New Zealand	Employee retention	*Antecedents*: perceived organisational support (POS) as dimension of employer attractiveness *Mediator*: organisational commitment
2	Bareket-Bojmel and Shuv-Ami (2019)	International Journal of Manpower	Israel	Turnover intention	*Antecedents:* organisational brand equity *Mediator*: organisational commitment
3	Bussin and Mouton (2019)	South African Journal of Economic and Management Sciences	South Africa	Compensation expectation, retention	*Antecedents:* employer brand perceptions
4	Deepa and Baral (2019)	Journal of Organisational Effectiveness-People and Performance	India	Employee engagement	*Antecedents*: different dimensions of employer brand – economic value (e.g., salary and compensation), development value (e.g., career growth opportunities, empowerment to take decisions, opportunities to develop new skills through training), social value (e.g., culture of supportive and encouraging colleagues, culture of supportive leadership), work value (periodic feedback on performance, adequate resources to perform on the job), employer reputation (organisation’s focus on environmental and CSR activities, organisation’s focus on high quality products and/or services, organisation’s focus on innovative products and/or services)
5^b^	Hamidizadeh and Fadardi (2019)	Human Systems Management	Iran	Employee retention, employee productivity	*Antecedents*: different dimensions of employer brand (economic value, development value, social value, work value and employer reputation) *Mediators*: organisational identity, job satisfaction, organisation commitment
6	Slatten et al. (2019)	BMC Health Services Research	Norway	Turnover intention, employee engagement, services quality provision	*Antecedents*: internal market-oriented cultures (IMOC), i.e. frontline-focused culture *Mediator*: organisational attractiveness
7^b^	Tumasjan et al. (2019)	Human Resource Management	Germany	Firm performance	*Antecedents*: employer brand orientation *Mediator*: positive affective climate (employees only)
8	Davies et al. (2018)	Journal of Organisational Effectiveness-People and Performances	United Kingdom	Employee engagement	*Antecedents*: employee views of corporate brand image *Mediators*: employee satisfaction *Moderators*: age, experience gender, whether the role involved customer contact
9	De Stobbeleir et al. (2018)	International Journal of Human Resource Management	Belgium	Employee absenteeism	*Antecedents*: internal employer brand perceptions (job content, career development, social atmosphere, financial rewards, and work–life balance)
10	Hoppe (2018)	Journal of Product and Brand Management	Germany	Brand citizen behaviour	*Antecedents*: perceived employer brand image *Mediators*: corporate brand identification
11	Kashyap and Verma (2018)	International Journal of Organisational Analysis	India	Employee turnover intention	*Antecedents*: different dimensions of employer brand (interest value, social value, development value, application value, economic value)
12	Matongolo et al. (2018)	Industrial and Commercial Training	Uganda	Talent retention	*Antecedents*: reward strategy, people oriented-ness, leadership and development
13	Mohamad et al. (2018)	International Food Research Journal	Malaysia	Employee retention	*Antecedents*: employer brand dimensions (organisation, individual, growth)
14	Sahu et al. (2018)	Leadership & Organisation Development Journal	India	Employees’ intention to leave, employer brand, psychological attachment	*Antecedents*: transformational leadership *Mediators*: employee engagement, employer branding
15	Ul Hadi and Ahmed (2018)	Administrative Sciences	Pakistan	Employee retention	*Antecedents*: different dimensions of employer brand (application, development, interest value) work-life-balance
16	Charbonnier-Voirin et al. (2017)	Canadian Journal of Administrative Sciences	Canada	Organisational identification, positive word-of-mouth	*Antecedents*: value congruence *Mediators*: employer brand
17	Guerrero and Challiol-Jeanblanc (2017)	Personnel Review	France	Turnover intention	*Antecedents*: perceived external prestige (PEP) *Moderator*: *ex ante* i-deals
18^b^	Urbancova and Hudakova (2017)	Economics & Sociology	Czechia	Employee retention, employee motivation, financial performance	*Antecedents*: knowledge continuity, talent management, age management, diversity management, Career Management *Mediator*: employer brand *Moderators*: business sector, majority ownership (national vs. international), size of organisation, number of employees, gender
19	Ardakani et al. (2016)	Iranian Journal of Management Studies	Iran	Human resource productivity (job satisfaction, turnover intention, organisational citizenship behaviour (OCB), job involvement)	*Antecedents*: Diversity Management *Mediators*: Organisational attractiveness, organisational justice, social identity
20	Kashyap and Rangnekar (2016b)	Global Business Review	India	Turnover intention	*Antecedents*: different dimensions of employer brand *Mediator*: trust
21	Kashyap and Rangnekar (2016a)	Review of Managerial Science	India	Employee turnover intention, employer brand	*Antecedents*: servant leadership, employer brand perception *Mediators*: level of trust employees placed in their leaders and employer brand perception
22	Tanwar and Prasad (2016)	Management Decision	India	Job satisfaction	*Antecedents*: different dimensions of employer brand (Training and development, reputation, diversity, work-life-balance, Corporate Social Responsibility CSR, organisational culture *Moderators*: gender
23^b^	Uen et al. (2015)	Asia Pacific Journal of Human Resources	Taiwan	Positive word-of-mouth by employees	*Antecedents*: employer brand management *Mediators*: organisational prestige (internal and external)
24	Dogl and Holtbrugge (2014)	International Journal of Human Resource Management	Multiple	Employee commitment	*Antecedents*: green strategy culture, green technology and products, green recruitment and evaluation, green communication *Moderators*: environmental reputation, cultural differences (developed vs. developing countries)
25	Hanin et al. (2013)	Psychologica Belgica	Belgium	Affective commitment	*Antecedents*: different dimensions of employer brand (employment experience and employment offering) *Mediators*: perceived organisational support and psychological contract violation
26	Ito et al. (2013)	Career Development International	Canada	Retrospective satisfaction, affective commitment, intention to search	*Antecedents*: employer brand attributes (pay, flexibility, security, development, promotion, values) *Moderators*: from entry to exit, age, between managers and staff
27	Van Hoye (2013)	Human Performance	Belgium	Positive or negative referrals	*Antecedents*: help job seekers find good-fitting jobs/avoid bad-fitting jobs, help the organisation find good-fitting employees/avoid bad-fitting employees, job satisfaction or dissatisfaction
28	Schlager et al. (2011)	Journal Of Services marketing	Multiple	Employees’ satisfaction and identification with the company	*Antecedents*: different dimensions of employer brand (economic, development, social, diversity and reputation value)
29	Davies (2008)	European Journal of Marketing	United Kingdom	Perceived differentiation, affinity, satisfaction and loyalty	*Antecedents*: different dimension of employer brand
30^b^	Lievens et al. (2007)	British Journal of Management	Belgium	Employee identification	*Antecedents:* perceived identity dimensions, construed external image dimensions

*^a^Studies are listed by the year of publication from latest to earliest, in alphabetical order.*

*^b^Study focused on both, potential and existing employees.*

### Key Characteristics of the Relevant Employee-Based Studies

We analyzed the key characteristics of each of the 48 quantitative studies, and since 38 of these, had been published since 2015, the issue of employer attractiveness as perceived by existing employees seems to be of increasing relevance in recent years. The main geographic sources of the empirical quantitative studies were India (*N* = 13), which mainly focused on the IT sector (Biswas and Suar, 2016; Sahu et al., 2018), followed by Belgium (*N* = 6), the United Kingdom (*N* = 3), and Germany (*N* = 3). Several studies (*N* = 8) were conducted in multiple countries, whose names were not always mentioned. In total, employee-based quantitative research was conducted in at least 21 countries. These 48 studies were published in 38 journals, but only two –the Journal of Organizational Effectiveness: People and Performance (*N* = 3) and Personnel Review (*N* = 3) – published more than two of the studies that were included in the sample. Of the 48 studies, 46 were published in journals with an Eigenfactor value [measuring the number of times articles from the journal published in the past five years have been cited in the Journal Citation Reports (JCR)] less than 0.90, indicating that articles in the journal has below-average influence. Research papers with an Eigenfactor value of 90 or higher were published by Slatten et al. (2019) and Trybou et al. (2014).

### Summary of the Employee-Based Empirical Studies on Employer Attractiveness

The quantitative studies identified as relevant for employer attractiveness as perceived by employees have been summarized as follows. Table 1 includes studies focusing on employer attractiveness or related concepts as outcomes (*N* = 18), while Table 2 includes studies that refer to employer attractiveness or related concepts, as antecedents or mediators for different factors of human productivity, such as commitment and/or turnover intentions (*N* = 27), or antecedents of word-of-mouth (WOM) marketing (*N* = 2), or firms’ performance (*N* = 1).

## Findings

A quantitative analysis of literature development on employee and applicant-based literature on employer attractiveness is summarized in Table 3. While literature on employer attractiveness has evolved over the past three decades, the first notion of the term *employer attractiveness* was developed by Turban and Greening (1997), who described it as the degree to which a respondent would personally seek an organization as an employer. Conversely, e*mployer branding* describes the actions undertaken by an organization to develop employer knowledge (Theurer et al., 2018). The outcome of brand building activities is the *employer brand*, that was first described by Ambler and Barrow (1996) as “the package of functional, economic and psychological benefits provided by employment and identified with the employing company” (p. 187). Literature acknowledges that employer branding tends to be often overshadowed by corporate external branding (Sengupta et al., 2015). *External branding* includes activities such as moral practices of leaders, and those related to corporate social responsibility (CSR) that influence the external perception of the employer brand, whereas *internal employer branding* aims at retaining or motivating employees to achieve organizations’ performance goals or fulfil their promises to the capital market.

**TABLE 3:** Quantitative analysis of literature development on employee- and applicants-based literature on employer attractiveness.

Empirical papers on employerattractiveness	I. Rise of employee-focus (2010-2019)													II. Laying the ground (1990-2009)													
Year	Total	Total	Oct 2019	2018	2017	2016	2015	2014	2013	2012	2011	2010	Total	2009	2008	2007	2006	2005	2004	2003	2002	2001	2000	1999	1997	1994	1992
Potential employees	227	169	21	34	20	28	21	8	13	6	8	10	58	2	8	7	3	5	8	8	6	4	3	1	1	1	1
**Employees**	***52***	***49***	***9***	***13***	***7***	***9***	***4***	***2***	***4***		***1***		***3***		***1***	***2***											
Antecedents	*18*	*17*	*2*	*3*	*4*	*4*	*2*	*1*	*1*				*1*			*1*											
Thereof outcomes	*30*	*28*	*7*	*8*	*3*	*4*	*1*	*1*	*3*		*1*		*2*		*1*	*1*											
Thereof qualitative	*4*	*4*		*2*		*1*	*1*																				
**Total**	**279**	**218**	**30**	**47**	**27**	**37**	**25**	**10**	**17**	**6**	**9**	**10**	**61**	**2**	**9**	**9**	**3**	**5**	**8**	**8**	**6**	**4**	**3**	**1**	**1**	**1**	**1**
% of employee-based papers	19%	22%	30%	28%	26%	24%	16%	20%	24%		11%		5%		11%	22%											
% of articles published on employer attractiveness	100%	78%	11%	17%	10%	13%	9%	4%	6%	2%	3%	4%	22%	1%	3%	3%	1%	2%	3%	3%	2%	1%	1%	0,4%	0,4%	0,4%	0,4%
% of articles published on employees compared to number of articles on employees	100%	94%	17%	25%	13%	17%	8%	4%	8%		2%		6%		2%	4%											
																											

Looking at the development of these studies, literature on employer attractiveness can be grouped into two phases: “Laying the ground (1990–2009)” and “Rise of employee-focus (from 2010).” During the first phase, although ground-breaking studies were published by Ambler and Barrow (1996) and Turban and Greening (1997), and the relevance of employer attractiveness for existing employees was noticed, yet the focus was on *potential* employees. Berthon et al. (2005) concluded that there was a high similarity between the employer brand and other concepts, such as internal marketing and employer branding, and also refer to existing employees. In the same study, Berthon et al. (2005) summarized their quantitative findings in a model that is based on a three-dimensional framework – functional, economic, and psychological – developed by Ambler and Barrow (1996). This model measures how attracted individuals are to their employers based on the following dimensions: economic value (compensation and benefits, job security, and opportunities for promotion), development value (recognition, self-worth, confidence, and future employment), interest value (exciting work environment, e.g., innovative products and services), social value (a fun-oriented and happy working environment, team atmosphere, etc.), and application value (opportunity to apply as well as teach others what was learned). Lievens and Highhouse (2003) and Lievens et al. (2007) established a different perspective of employer attractiveness that distinguished between the two aspects of organizations’ employer branding: (i) symbolic – subjective, abstract, and intangible attributes, and (ii) instrumental – objective, physical, and tangible attributes. The drivers of how individuals perceive different attributes of employment offerings are based on *fit theories* (Van Hoye and Turban, 2015), labeled as the *person-organization-fit* (POF) and *person-environment-fit* (PEF). The POF theory stresses that congruence between people’s characteristics (values, motives, skills, and experience) and organizations’ characteristics (structure, tasks, technology, organizational values, and climate) are crucial for the success of the people working within these organizations, as well as the organizations themselves (Kristof, 1996; Van Vianen, 2000). The PEF perspective proposes that applicants are more attracted to employers, who offer characteristics compatible with their own characteristics (Kristof-Brown et al., 2005). The literature shows that employees who fit well with recruiting organizations, exhibit higher levels of organizational identification, productivity, co-worker and supervisor satisfaction, job satisfaction, and lower turnover rates (Kristof-Brown et al., 2005). From the perspective of potential employees, employer attractiveness measures are used to predict the organizational pursuits of potential employees (Highhouse et al., 2003). However, as pointed out by Cable and Turban (2003), potential employees lack the experience of working in the target organization yet, and their perceptions might not provide complete and accurate information about the employment experience. This suggests that there is a difference in how organizational actions are perceived by potential and existing employees. There is also a difference in potential outcomes. While the measured outcomes of employer attractiveness are application intentions or job pursuits for potential employees, the outcomes are employee retention, employee engagement, or positive WOM for existing employees. During the first phase – “Laying the ground (1990–2009)” – out of a total of 61 empirical studies on employer attractiveness, only three studies, that focused on employees, at least to some extent, were published (Lievens, 2007; Lievens et al., 2007; Davies, 2008).

During the second phase – “Rise of employee-focus (from 2010)” – employees became increasingly prominent for both antecedents (*N* = 17) and outcomes (*N* = 28) of employer attractiveness. Schlager et al. (2011) indicated that certain aspects of employer branding – renumeration and development – increase employees’ satisfaction and identification with their organizations. Among the studies on the different drivers of employee-perceived employer attractiveness, those of Ambler and Barrow (1996), Backhaus and Tikoo (2004) and Berthon et al. (2005) were further developed and adopted for employee perspectives. For example, based on Berthon et al. (2005)’s model, Tanwar and Prasad (2017) developed a 5-scale-model, specifically for employees, for testing the dimensions of a healthy work atmosphere; training and development; work-life balance; ethics and CSR; and compensation and benefits.

Theurer et al. (2018) reviewed the existing literature, along with the guiding theoretical construct of the marketing-based brand equity theory, and integrated the different perspectives of research conducted over the last few decades, into an innovative employer branding value chain model. Although their model does not explicitly focus on employer attractiveness, in different constellations, its building blocks have been used for employer attractiveness research as follows: (1) employer knowledge development and investment – what companies can do, (2) applicant/employee mindset – what applicants/employees think, feel, and do, (3) organizational performance and competitive advantage – what companies get, and (4) financial market performance and shareholder value – monetary value of employer branding.

## Integration

**FIGURE 2:**
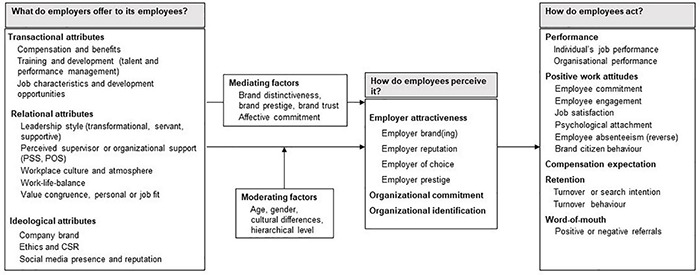
Antecedents and consequences of employer attractiveness as perceived by employees of that organisation.

We integrated the factors identified during our research that influence employee-related employer attractiveness (e.g., compensation or leadership style), as well as those, that mediate (e.g., brand trust) or moderate (e.g., age, hierarchy level) that relationship. We also incorporated the outcomes of employer attractiveness (e.g., performance, retention), including relationships where employer attractiveness is found to be a mediator. The findings were structured based on the Inputs-Mediators-Outcomes model (Figure 2). It should be noted that in some of the more recent studies, instead of employer attractiveness, its different attributes are related either to organizational commitment or organizational identification (Arasanmi and Krishna, 2019; Hamidizadeh and Fadardi, 2019). Hence, we suggest that future research should focus on the potential differences and similarities between employer attractiveness, organizational commitment, and organizational identification.

### Antecedents of Employer Attractiveness

The few studies that have solely focused on internally perceived employer attractiveness provide evidence that several factors such as compensation or leadership style influence how employees see their employers. To distinguish the different antecedents influencing internally perceived employer attractiveness, we have drawn on the theory of Anticipatory Psychological Contracts (APC), a theoretical framework capturing potential employees’ perceptions of firms’ employment offerings, that has been used by Moser et al. (2017) to describe dimensions of employer attractiveness for potential employees in new ventures. Moser et al. (2017) used the APC theory to “demonstrate how and to what extent their employment offering is distinct from that of other new ventures competing for members of the same talent pool” (p. 591). This is also in line with the research done by Lievens and Slaughter (2016), who confirmed that job seekers use a variety of employment attributes to develop an image of different employment options.

Theorizing on the nature of the elements of firms’ employment offerings, the APC theory proposes that applicants base their job decisions primarily on three types of employer attributes: *ideological* (e.g., culture), *transactional* (e.g., monetary or other tangible compensation), and *relational* (e.g., characteristics of the employer-employee relationship, such as social support provided by the management or colleagues; Moser et al., 2017). Since not only do potential employees decide on which jobs to take, but existing employees also regularly decide whether to stay on with an employer or quit, we argue that the attributes offered by employers can also be grouped using the APC theory. Out of the 48 quantitative studies reviewed, while 18 focused on either employer brand attractiveness or brand commitment as dependent variable, the remaining 30 researched on outcomes other than employer attractiveness, such as employee commitment and job satisfaction, or relationships where employer attractiveness was found to be a mediator. Our model too, included relevant antecedents.

In the following section, we have grouped the findings of studies into different attribute categories based on the APC model used by Moser et al. (2017), which differentiates employment offerings into transactional, relational, or ideological attributes.

#### Transactional Attributes

Transactional attributes describe the tangible factors offered by employers. Based on the studies identified during our research, we found that compensation and benefits; training and development; and job characteristics and development opportunities, qualify as transactional attributes. Other factors like facilities; office location and infrastructure; or the possibilities of intrapreneurship, have also been confirmed as relevant (Chawla and Lenka, 2015; Sengupta et al., 2015).

##### Compensation and Benefits

Empirical studies have resulted in controversial outcomes. Tanwar and Prasad (2017) discovered that compensation and benefits were the least influential dimensions of the employer brand, whereas Roongrerngsuke and Liefooghe (2013) discovered, during their research in China, India, and Thailand, that the importance of compensation and benefits were high, for all three generations studied (Baby Boomers, Generation X, Generation Y). Lievens (2007) identified pay and benefits as factors with the highest importance for perceived employer attractiveness, among employees of the Belgian Army. Schlager et al. (2011) confirmed that economic attributes (e.g., compensation) positively influence job satisfaction, although its positive correlation with organizational identification could not be confirmed. Summarizing the findings, it can be said that the relevance of compensation for the perception of employer attractiveness, in the eyes of employees, depends on the context, and there are no clear views that can be generalized.

##### Training and Development

The importance of opportunities for training and development have been studied numerous times, either together or with other attributes (Lievens, 2007; Schlager et al., 2011; Roongrerngsuke and Liefooghe, 2013; Tanwar and Prasad, 2016, 2017), or as specific aspects of training and development (Vnouckova et al., 2018). Employees are interested in improving and developing their skills for future job positions, either within the same company, or with other companies (Tanwar and Prasad, 2017), which leads companies to invest significant budgets for employees’ training and development.

##### Job Characteristics and Development Opportunities

Opportunities for enhancing career growth and building self-esteem also appear to be common predictors of perceived employer attractiveness (Trybou et al., 2014; Nayak and Suhan, 2017). Therefore, realistic job previews were found to be important for potential as well as existing employees (Biswas and Suar, 2016). Among other factors, Sengupta et al. (2015) confirmed the relationship between “career potential value factors” like skill utilization for internally perceived employer attractiveness.

#### Relational Attributes

Relational attributes describe factors that determine the relationships between other members of the organization and employees. During our research, we identified the following factors as predictors of internally perceived employer attractiveness:

#### Leadership Style

The influence on leadership styles has been studied from two different angles in relation to employer brands, by investigating the impact of transformational or servant leadership. What all studies have in common is evidence, that leadership plays a key role in influencing and projecting a positive organizational image. The most recent study on the influence of leadership was published by Sahu et al. (2018). It provided evidence that transformational leadership is an antecedent of the employer brand, which is mediated by employee engagement. Therefore, this study explored the influence of engaged employees in building employer branding. The second study in leadership, conducted by Kashyap and Rangnekar (2016a), indicates that “servant style of leadership if followed by organizational leaders, may prove to be an effective tool to portray a positive organizational image amongst potential and existing employees” (p. 454). In both studies, the ultimate outcome in relation to existing employees would be the reduction of employees’ turnover intentions through a strong employer brand.

#### Perceived Supervisory or Organizational Support

Perceived Supervisory Support (PSS) and Perceived Organizational Support (POS) determine employer attractiveness as perceived by employees, since as representatives of the organization, supervisors drive organizational behavior (App and Buettgen, 2016). The study revealed that POS does not directly affect brand commitment, but is mediated by the brand’s distinctiveness, prestige, and trust, and also confirms the positive impact of PSS on POS. This means that if employees perceive their supervisors as being engaged in maintaining their work resources, they believe such behaviors as being representative of the organization. In the same context, another research was conducted by Biswas and Suar (2016), which confirmed that POS, together with organizational trust and leadership of top management is positively correlated with employer attractiveness.

#### Workplace Culture and Atmosphere

The complex field of workplace culture and atmosphere has been an ongoing theme in employer attractiveness research. Reis et al. (2017) identified workplace authenticity – commonly described as knowing one’s self and acting accordingly – as an attribute of employer attractiveness. The results show that respondents rated authenticity more highly than the other dimensions – economic, development, interest, social, and application values – included in the employer attractiveness scale. Although the mean scores of authenticity were always higher, statistically significant differences were found exclusively for comparisons between authenticity and interest, social, and application values. The differences between authenticity, economic value, and development value were not statistically significant. Tanwar and Prasad (2017) determined five dimensions of the employer brand as perceived by existing employees: training and development; healthy work atmosphere; work-life balance; ethics and CSR; and compensation and benefits, and also found a “healthy work atmosphere” to be one of the most significant dimensions influencing the employer brand. De Waal (2018) confirmed that a possible way of creating an attractive organization is by transforming the workplace into a high-performing organization.

#### Work-Life Balance

Along with workplace culture and atmosphere, Tanwar and Prasad (2017) found work-life balance to be the third most important dimension that influences the employer brand, indicating that employees prefer to work in organizations that provide flexible work hours and work from home facilities. Maurya and Agarwal (2018) identified work-life balance as one of the drivers, not only in relation to employer attractiveness, but especially for retaining talent in organizations.

#### Value Congruence, Personal, or Job Fit

As described above, an organization’s attractiveness highly depends on applicants’ individual characteristics (Kristof-Brown et al., 2005). This was picked up by Charbonnier-Voirin et al. (2017), thereby confirming the impact of value congruence – match between organizational values and employees’ values – on organizational identification and positive word-of-mouth, mediated by employer brands. The relevance of certain personality traits were the focus of two recent empirical studies that used crowdsourcing (Dabirian et al., 2019; Robertson et al., 2019). Although Robertson et al. (2019) did not explicitly study personality traits as being moderators, the crowdsourcing analysis revealed that different personality traits led to different rankings of employer brand personalities, which indicates that personality traits impact how employees perceive employer attractiveness.

#### Ideological Attributes

Ideological attributes (i.e., commitment to a valued cause, Moser et al., 2017) describe the intangible aspects of an organization. During our research, we identified the following factors as predictors of internally perceived employer attractiveness:

##### Company Brand

Dogl and Holtbrugge (2014) conducted an empirical study among 215 firms in China, Germany, India, and the United States of America (United States). Its results revealed that green strategies, culture, products, and communication positively influence the environmental reputation of the company as an employer, and in turn, employee commitments with “green,” that refer to responsible environmental aspects. Since this study cannot be generalized, we later identified business aspects, such as corporate mission, strategy, products, and impact on employer attractiveness as perceived by employees, as one of the key areas for the agenda of future research.

##### Ethics and Corporate Social Responsibility

This factor was found to be the fourth most important dimension that influences the employer brand in Tanwar and Prasad (2017)’s empirical study. However, besides this study and those by Dogl and Holtbrugge (2014) and Biswas and Suar (2016), which have been previously described, not much research with existing employees has been done, although this topic has mostly been researched with potential employees (Klimkiewicz and Oltra, 2017).

##### Social Media Presence and Reputation

The role of social media is a rather recent development in the literature on employer attractiveness (Dabirian et al., 2017; Robertson et al., 2019). Both studies confirm the relevance of social media presence and reputation (word-of-mouth) for existing employees.

### Moderators

Several variables that moderate the relationship between the different input variables and employer attractiveness brand were identified.

#### Gender

Studies by Reis et al. (2017) and Tanwar and Prasad (2016) have identified gender as a powerful moderator in the context of employer attractiveness. To cite an example, Tanwar and Prasad (2016), who investigated the effect of different employer brand dimensions on job satisfaction in the Indian IT sector, using gender as the moderator discovered that males are more affected by organizational reputation, training, and development, as against females, who are more affected by work-life balance, CSR, and organizational culture. Although the study does not reveal the reasons for the differences in perceptions among males and females, the authors believe that an explanation for this finding could be the different orientations toward work values. Reis et al. (2017) revealed that females give more importance to authenticity in the workplace than males.

#### Age

Roongrerngsuke and Liefooghe (2013) discovered during their research in China, India, and Thailand that the generations, whom they studied, considered compensation and benefits as important. Across the three countries, competitive rewards were mentioned as the most important factor (78.4%) for baby boomers (those born between 1946 and 1965), whereas the top two attractive factors for Generation X (those born between 1966 and 1979), were the company’s brand image (57.9%) and competitive rewards (76.4%) and for Generation Y (born between 1980 and 1999) too, they were competitive rewards (79.3%) and company’s brand image (51.5%).

#### Cultural Differences

Dogl and Holtbrugge (2014) discovered through their corporate environmental responsibility study in China, Germany, India, and the United States, that assumed cross-national differences in perceptions were immaterial, e.g., for green strategies and culture; green technology and products, etc. The authors reasoned that in China and India, due to significant increases in wages for the researched cohort (employees of different corporations), once basic needs are satisfied, considerations relating to environmental issues become more relevant. Thus, in general, while cultural differences prevail, globalization also leads to a convergence of cultural values in the business context, especially for globally operating firms.

#### Hierarchy Level

Reis et al. (2017) revealed that authenticity in the workplace was contingent on employees’ gender, age, and hierarchical levels. In particular, the higher the hierarchy level and the older the employees (male or female), the greater was their appreciation for authenticity in the workplace.

### Mediators

Several studies have identified relationships that were mediated by different variables. Although it is impossible to generalize such mediating relationships to other antecedents, it seems to be a repetitive theme that individual perceptions play a role in how individuals perceive certain attributes of employer attractiveness. App and Buettgen (2016), found that the relationship between POS/PSS and employer brand commitment is mediated by perceived brand prestige. The mediating role of affective commitment toward employer brands was also confirmed by Fernandez-Lores et al. (2016).

### Consequences of Employer Attractiveness

The outcomes of internally perceived employer attractiveness can be positive word-of-mouth, retention, positive work attitudes, compensation expectations, or performance, which are discussed in this section. In several studies, employer brand(ing) or attractiveness was identified as a mediator. The consequences of such a constellation are also included here.

#### Performance

Employees’ performance as a positive outcome of employer attractiveness can be seen two-fold. On an individual level, Hamidizadeh and Fadardi (2019) found that the different dimensions of employers’ brands increase employee productivity. On an organizational level, Tumasjan et al. (2019), and Urbancova and Hudakova (2017), confirmed the positive aspects of employers’ brand orientation or branding on firms’ performance (financial).

#### Positive Work Attitudes

Employees’ commitment and engagement, job satisfaction, psychological attachment, absenteeism (reverse), and brand citizen behaviors have been studied as outcomes of internally perceived employer attractiveness in numerous cases. The study by Schlager et al. (2011) was one of the first, to draw a conclusion on the importance of delivering value to employees, that would enhance their levels of satisfaction and result in their identification with their employers, since it leads to positive business consequences (customers’ experiences in employee–customer interactions). Taking this further, Tanwar and Prasad (2016) focused on the key dimensions of employer branding and empirically examined the impact of its different dimensions on job satisfaction. Sahu et al. (2018) discovered that transformational leadership had a positive influence on employees’ intentions to leave, and was mediated by both, employer engagement and employer branding.

#### Compensation Expectations

Employees’ compensation expectations are a critical factor in employee engagement. Bussin and Mouton (2019)’s study indicates that there is a correlation between employees’ perceptions of employer branding in their own organizations and their willingness to work for lower salary and benefits, which also increases.

#### Retention

Aspects of retention (turnover or search intentions and turnover behavior) are the most studied consequences of employer attractiveness or its antecedents. The results of Kashyap and Rangnekar (2016a)’s study indicate that employer brand perceptions significantly mediate the relationship between servant leadership styles and employees’ turnover intentions. These findings indicate that servant style leadership followed by leaders, helps in creating and reinforcing a strong employer brand image in the minds of existing employees, which in turn, influences their decision to extend their association with their organizations. Other studies too, have indicated that POS or organizational brand equity positively influence employee retention or decrease their turnover intentions, mediated by organizational commitment (Arasanmi and Krishna, 2019; Bareket-Bojmel and Shuv-Ami, 2019).

#### Word-of-Mouth or Referrals

While research on employer attractiveness and word-of-mouth has been mainly driven by Van Hoye and Lievens (2007), Van Hoye and Lievens (2009) their focus has been on job seekers, and not on employees. Recently, four studies conducted research on the perspectives of employees. Van Hoye (2013) confirmed that the desire to help job seekers find good-fitting jobs and organizations to find good-fitting employees, as well as individuals’ job satisfaction, correlate with positive or negative referrals. In light of new technological capabilities, Dabirian et al. (2017) conducted a study on employer branding, by structuring seven value propositions for employer branding – social, interest, application, development, economic, management, and work-life balance – into true motivators and hygiene factors. Charbonnier-Voirin et al. (2017) and Uen et al. (2015) also found a positive correlation between employer attractiveness and positive word-of-mouth.

## Discussion and Future Research Directions

### Discussion

This study’s objective was to summarize and integrate the evidence on what drives internally perceived employer attractiveness as well as its consequences, particularly when employees perceive the organizations they are working for, as attractive employers. First, the main finding relates to the availability of only a few studies, that have researched employer attractiveness with actual employees. However, what can now be said is, that there is a body of evidence that lends some support to the view that high levels of attractiveness are beneficial for employers, and that aspects of what might be considered good management and leadership practices may serve to raise attractiveness levels to increase retention or decrease turnover behaviors.

Second, since only 18 studies are available within the database of WoS (Table 1), that focus on antecedents of employer attractiveness with employees, there is limited evidence on which factors (e.g., good management practices) influence internally perceived employer attractiveness. Some evidence can be drawn from studies, wherein employer attractiveness or related concepts are mediators (Table 2). In addition, while some factors – training and development – have been studied multiple times, for others (e.g., value congruence), there is only one study in a very specific context that might not be valid for generalization. From our perspective, it is fair to say that attributes of employees’ internally perceived employer attractiveness have not been studied in the same breadth and depth as that of jobseekers.

Third, the 30 studies (Table 2) on outcomes of perceived employer attractiveness (or related concepts) focus to a large extent on turnover intentions or retention. Word-of-mouth, one of the key sources of information, especially in the digital age, has been occasionally studied with employees, but by far, not to the extent that it has been studied with potential employees. There is hardly any evidence on what factors drive positive word-of-mouth in relation to perceived employer attractiveness.

Fourth, based on the antecedents and outcomes, it can be concluded that employer attractiveness, organizational commitment, and organizational identification are somewhat related concepts since their constructs appear to have similar antecedents and consequences. However, as on now, this is an assumption that needs to be verified.

Fifth, although research on existing employees has been picking up during the last few years, regional and industry focus is very limited. Among the few existing studies, most have been conducted in India’s IT sector, which provides good insights on organizations with strong demands for labor and high turnover rates. However, it remains unclear whether their results can be generalized, or if in less dynamic working environments, the perceptions of employees are different. Moreover, the fact that only a few empirical studies have been published in top management journals indicates the immaturity of the field.

Despite our careful identification and comprehensive integration of extant literature, there were limitations that need to be acknowledged. First, we reviewed only journal publications in English, and omitted book chapters, books and other scientific contributions not included in the database of WoS. Second, having restricted our research to certain search terms, we cannot, therefore, be sure of having captured all relevant studies, although our search should have ensured that any study with the word “employer attractiveness” in the abstract or title was captured. Third, we decided to omit from our literature review any publications, whose focus was either too narrow or different on the basis of our academic judgment, which could have potentially influenced our findings.

### Future Research Directions

Our synthesis of evidence has highlighted important gaps in knowledge, revealing that out of the 279 empirical studies on employer attractiveness, 227 focused on potential employees, whereas only 52 focused to some extent on existing employees. Hence, most of the knowledge accumulated over the past 20 years is based on the external perspective of employer brands. When Ambler and Barrow (1996) published their first study titled “The Employer Brand,” and with that, laid the base for future research on the topic, they had already pointed out that the employer brand is highly relevant for both, potential and existing employees. Therefore, the field of employer attractiveness through the eyes of its employees, provides a high potential for future research, given the fact, that there are indications that potential employees, especially students’ perceptions of employer attractiveness will be different from those of experienced employees (Lievens, 2007; Reis et al., 2017). Some of the identified gaps are described below.

#### Antecedents of Employer Attractiveness

We need to have a better understanding of the factors that influence internally perceived employer attractiveness, especially when it comes to variables, such as products or industry, that are more on the business side of an organization. The research by Roongrerngsuke and Liefooghe (2013) and Lievens (2007) indicate to some extent, that the brands of companies, their symbolic attributes, as well as attributes beyond human resource activities and leadership, are relevant for the perception of attractiveness. Especially in times of industry disruption (e.g., from fuel cars to electronic vehicles, or from banking counters to apps), we need to understand more about how such factors impact the employer brands of potential as well as existing employees, but mostly, existing employees, since most organizations rely on the fact that their employees will help them to manage the change. A current study by Deepa and Baral (2019) found that among the 40 employer brand attributes perceived by current employees, “Organizations’ focus on innovative products and services” ranked #11, while “Organization’s focus on environmental and CSR activities” ranked #35. Furthermore, Moser et al. (2017)’s study indicated that the legitimacy of founders in the context of new ventures, had a significant positive effect on organizational attractiveness for job seekers. Since traditional companies are striving to become more innovative, so as to attract and retain talent with an entrepreneurial mindset, we need to have a better understanding of the extent to which, having an entrepreneurial direct boss or CEO would increase employer attractiveness. Are there entrepreneurial “pockets” within an organization? Does it make sense for managements to create such pockets by evaluating the entrepreneurial spirit of candidates? Are employees more attracted to the company brand, industry, CEO of a company, or direct boss? The results of such research would help us to focus better on our HR activities, as well as help organizations to achieve their strategic goals.

#### Influence of Moderators on the Attractiveness of Employers

Since only a few moderators were studied with regard to existing employees, we do not have enough knowledge on how the activities of internal employer branding are perceived in different contexts. For example, we considered the role of moderators in corporate communications related to employment offers, as being an important field of research. Milman and Dickson (2014) identified corporate communications as one of the key antecedents of employee retention in their study on theme parks in the United States, which tested several antecedents, such as work environment or training opportunities, that also influence employer attractiveness. We need to accumulate more knowledge on how such communications are perceived to better understand how the phenomenon of employer attractiveness is formed and influenced through different communication channels at different hierarchical levels, genders, generations, or years with an organization.

#### Specific Organizational Settings

Further studies that focus on internally perceived employer attractiveness, or longitudinal studies that examine the impact of initiatives aimed at changing internally perceived attractiveness would also serve to further develop this field. To the best of our knowledge, none of the studies on internally perceived employer attractiveness have examined interventions, such as training and development programs, or specifically focused on raising internally perceived employer attractiveness, which represents a significant gap in our knowledge. It would be useful to gain further insights into which interventions have the most impact, and under what conditions. Studies that apply and contextualize more generic frameworks relating to employer attractiveness to different organizational settings would also be welcome since the studies included were partially conducted in a very unique setting like the Belgian Army.

#### Longitudinal Studies

Many companies are currently redefining their business models through mergers and acquisitions, and its consequence will be the addition of new segments or product lines, which will have a major impact on how an organization is perceived as an employer by current employees and employees that are acquired, or those, who will be leaving the organization due to a carve-out. A study of antecedent variables in such contexts would enrich the understanding of employer attractiveness and provide managers with crucial information.

#### Comparing Levels of the Research Unit and Target Groups

Further studies that investigate attributes at different levels – individual, team, and organizational – would shed additional light on the concept of internally perceived employer attractiveness. Since existing research has been conducted at the individual level, it has not considered how attractiveness levels within and across teams could vary. It would also be useful to acquire additional knowledge about whether people are more attracted by their jobs, work teams, organizations, or professions. What role does social capital, that employees have built up during their career, play in the perception of employer brands? What is more important for retention: job, team, or organizational attractiveness? Since only one study has compared existing and potential employees in the special setup of the Belgian Army (Lievens, 2007), we recommend more comparative research that would distinguish between potential and actual applicants, as well as existing employees. This would enhance organizations’ targeting of their audiences, according to their needs.

As an area for research and practice, employer attractiveness as perceived by employees, continues to show significant promise. There is much scope for further research that would seek to develop and extend current conceptualizations and theorizations of attractiveness through investigations that take greater account of existing employees’ perceptions.

### Implications for Managers and Organizations

Looking at the challenges faced by certain industries or regions related to the shortage of skilled labor, keeping employees attracted to organizations as employers, becomes even more relevant for the future profitability and growth of organizations. With higher transparency in social media, e.g., LinkedIn, what happens “for real” inside organizations becomes more and more transparent. While the signaling theory has been the main driver for discussions on employer branding for the past 20 years, what if “signaling” is replaced by “knowing” what takes place inside organizations? This would mean that employees become the main ambassadors of organizations, and employer branding will increasingly be taken over by the communications or non-communications of the people working within organizations. This would mean that employer branding needs to be targeted more toward employees, and less toward potential employees, which at present is rare.

## Data Availability Statement

The original contributions presented in the study are included in the article/supplementary material, further inquiries can be directed to the corresponding author.

## Author Contributions

AD is a PhD candidate, main author of the submitted manuscript, responsible for identifying relevant articles under the supervision of SK, who has expertise in literature review writing, did the initial analysis of the manuscript, and wrote the initial draft. SK, KK, and EL are PhD supervisors and helped to develop the manuscript toward the final submission. All authors contributed to the article and approved the submitted version.

## Conflict of Interest

The authors declare that the research was conducted in the absence of any commercial or financial relationships that could be construed as a potential conflict of interest.

## Publisher’s Note

All claims expressed in this article are solely those of the authors and do not necessarily represent those of their affiliated organizations, or those of the publisher, the editors and the reviewers. Any product that may be evaluated in this article, or claim that may be made by its manufacturer, is not guaranteed or endorsed by the publisher.

## References

[B1] Ambler T. Barrow S. (1996). The employer brand. *J. Brand Manag.* 4 185–206.

[B2] App S. Buettgen M. (2016). Lasting footprints of the employer brand: can sustainable HRM lead to brand commitment? *Employee Relat.* 38 703–723. 10.1108/er-06-2015-0122

[B3] Arasanmi C. N. Krishna A. (2019). Employer branding: perceived organisational support and employee retention - the mediating role of organisational commitment. *Industrial Commer. Training* 51 174–183. 10.1108/ict-10-2018-0086

[B4] Ardakani M. S. Abzari M. Shaemi A. Fathi S. (2016). Diversity management and human resources productivity: mediating effects of perceived organisational attractiveness, organisational justice and social identity in Isfahan’s steel industry. *Iran. J. Manag. Stud.* 9 407–432.

[B5] Bareket-Bojmel L. Shuv-Ami A. (2019). The brand is my workplace. *Int. J. Manpow.* 40 818–833. 10.1108/ijm-07-2017-0176

[B6] Backhaus K. Tikoo S. (2004). Conceptualizing and researching employer branding. *Career Dev. Int.* 9 501–517.

[B7] Berthon P. Ewing M. Hah L. L. (2005). Captivating company: dimensions of attractiveness in employer branding. *Int. J. Advert.* 24 151–172. 10.1080/02650487.2005.11072912

[B8] Biswas M. K. Suar D. (2016). Antecedents and consequences of employer branding. *J. Bus. Ethics* 136 57–72. 10.3389/fpsyg.2022.859614 35369242 PMC8972195

[B9] Bussin M. Mouton H. (2019). Effectiveness of employer branding on staff retention and compensation expectations. *S. Afr. J. Econ. Manag. Sci.* 22 a2412.

[B10] Cable D. M. Turban D. B. (2003). The value of organisational reputation in the recruitment context: a brand-equity perspective. *J. Appl. Soc. Psychol.* 33 2244–2266. 10.1111/j.1559-1816.2003.tb01883.x

[B11] Charbonnier-Voirin A. Poujol J. F. Vignolles A. (2017). From value congruence to employer brand: impact on organisational identification and word of mouth. *Can. J. Adm. Sci.* 34 429–437. 10.1002/cjas.1379

[B12] Chawla S. Lenka U. (2015). A study on learning organisations in Indian higher educational institutes. *J. Workplace Learn.* 27 142–161. 10.1108/jwl-07-2014-0052

[B13] Dabirian A. Kietzmann J. Diba H. (2017). A great place to work!? understanding crowdsourced employer branding. *Bus. Horiz.* 60 197–205.

[B14] Dabirian A. Paschen J. Kietzmann J. (2019). Employer branding: understanding employer attractiveness of IT companies. *IT Prof.* 21 82–89. 10.1109/mitp.2018.2876980

[B15] Davies G. (2008). Employer branding and its influence on managers. *Eur. J. Mark.* 42 667–681. 10.1108/03090560810862570

[B16] Davies G. Mete M. Whelan S. (2018). When employer brand image aids employee satisfaction and engagement. *J. Organ. Eff. People Perform.* 5 64–80. 10.1108/joepp-03-2017-0028

[B17] De Stobbeleir K. E. M. De Clippeleer I. Caniëls M. C. J. Goedertier F. Deprez J. De Vos A. (2018). The inside effects of a strong external employer brand: how external perceptions can influence organisational absenteeism rates. *Int. J. Hum. Res. Manag.* 29 1–31.

[B18] De Waal A. (2018). Increasing organisational attractiveness: the role of the HPO and happiness at work frameworks. *J. Organ. Eff. People Perform.* 5 124–141. 10.1108/joepp-10-2017-0080

[B19] Deepa R. Baral R. (2019). Importance-performance analysis as a tool to guide employer branding strategies in the IT-BPM industry. *J. Organ. Eff. People Perform.* 6 77–95. 10.1108/joepp-04-2018-0024

[B20] Dogl C. Holtbrugge D. (2014). Corporate environmental responsibility, employer reputation and employee commitment: an empirical study in developed and emerging econo-mies. *Int. J. Hum. Res. Manag.* 25 1739–1762. 10.1080/09585192.2013.859164

[B21] Fernandez-Lores S. Gavilan D. Avello M. Blasco F. (2016). Affective commitment to the employer brand: development and validation of a scale. *BRQ Bus. Res. Q.* 19 40–54. 10.1016/j.brq.2015.06.001

[B22] Guerrero S. Challiol-Jeanblanc H. (2017). Ex ante i-deals, perceived external prestige and turnover intentions. *Pers. Rev.* 46 1199–1212. 10.1108/pr-10-2015-0271

[B23] Hamidizadeh A. Fadardi M. M. (2019). The brand of a university as an employer. *Hum. Sys. Manag.* 38 73–86.

[B24] Hanin D. Stinglhamber F. Delobbe N. (2013). The impact of employer branding on employees: the role of employment offering in the prediction of their affective commitment. *Psychol. Belg.* 53 57–83. 10.5334/pb-53-4-57

[B25] Highhouse S. Lievens F. Sinar E. F. (2003). Measuring attraction to organisations. *Educ. Psychol. Meas.* 63 986–1001.

[B26] Hoppe D. (2018). Linking employer branding and internal branding: establishing perceived employer brand image as antecedent of favourable employee brand attitudes and behaviours. *J. Prod. Brand Manag.* 27 452–467. 10.1108/jpbm-12-2016-1374

[B27] Ito J. K. Brotheridge C. M. McFarland K. (2013). Examining how preferences for employer branding attributes differ from entry to exit and how they relate to commitment, satisfaction, and retention. *Career Dev. Int.* 18 732–752. 10.1108/cdi-05-2013-0067

[B28] Kashyap V. Rangnekar S. (2016a). Servant leadership, employer brand perception, trust in leaders and turnover intentions: a sequential mediation model. *Rev. Manag. Sci.* 10 437–461. 10.1007/s11846-014-0152-6

[B29] Kashyap V. Rangnekar S. (2016b). The mediating role of trust: investigating the relationships among employer brand perception and turnover intentions. *Glob. Bus. Rev.* 17(Suppl.) 64S–75S.

[B30] Kashyap V. Verma N. (2018). Linking dimensions of employer branding and turnover intentions. *Int. J. Organ. Anal.* 26 282–295. 10.1108/ijoa-03-2017-1134

[B31] Klimkiewicz K. Oltra V. (2017). Does CSR enhance employer attractiveness? the role of millennial job seekers’. attitudes. *Corp. Soc. Responsib. Environ. Manag.* 24 449–463. 10.1002/csr.1419

[B32] Korn Ferry Study (2018). *Future of Work: The Global Talent Crunch.* 4. Download available Online at here: https://dsqapj1lakrkc.cloudfront.net/media/sidebar_downloads/FOWTalentCrunchFinal_Spring2018.pdf (accessed October 21, 2020).

[B33] Kristof A. L. (1996). Person-organisation fit: an integrative review of its conceptualizations, measurement and implications. *Pers. Psychol.* 49 1–49. 10.1111/j.1744-6570.1996.tb01790.x

[B34] Kristof-Brown A. L. Zimmerman R. D. Johnson E. C. (2005). Consequences of individuals’ fit at work: a meta-analysis of person-job, person-organisation, person-group, and person-supervisor fit. *Pers. Psychol.* 58 281–342. 10.1111/j.1744-6570.2005.00672.x

[B35] Lievens F. (2007). Employer branding in the Belgian Army: the importance of instrumental and symbolic beliefs for potential applicants, actual applicants, and military employees. *Hum. Res. Manag.* 46 51–69. 10.1002/hrm.20145

[B36] Lievens F. Highhouse S. (2003). The relation of instrumental and symbolic attributes to a company’s attractiveness as an employer. *Pers. Psychol.* 56 75–102. 10.1111/j.1744-6570.2003.tb00144.x

[B37] Lievens F. Slaughter J. E. (2016). Employer image and employer branding: what we know and what we need to know. *Ann. Rev. Organ. Psychol. Organ. Behav. Contents* 3 407–440. 10.13075/mp.5893.00802 31070604

[B38] Lievens F. Van Hoye G. Anseel F. (2007). Organisational identity and employer image: towards a unifying framework. *Br. J. Manag.* 18 S45–S59.

[B39] Matongolo A. Kasekende F. Mafabi S. (2018). Employer branding and talent retention: perceptions of employees in higher education institutions in Uganda. *Industrial Commer. Training* 50 217–233. 10.1108/ict-03-2018-0031

[B40] Maurya K. K. Agarwal M. (2018). Organisational talent management and perceived employer branding. *Int. J. Organ. Analysis* 26 312–330. 10.1108/ijoa-04-2017-1147

[B41] Milman A. Dickson D. (2014). Employment characteristics and retention predictors among hourly employees in large US theme parks and attractions. *Int. J. Contemp. Hosp. Manag.* 26 447–469. 10.1108/IJCHM-04-2013-0178

[B42] Mohamad S. F. Sidin S. M. Dahlia Z. Boo H. C. Ho J. A. (2018). Conceptualization of employer brand dimensions in Malaysia luxury hotels. *Int. Food Res. J.* 25 2275–2284.

[B43] Mokina S. (2014). Place and role of employer brand in the structure of corporate brand. *Econ. Sociol.* 7 136–148. 10.14254/2071-789x.2014/7-2/11 33338867

[B44] Moser K. J. Tumasjan A. Welpe I. M. (2017). Small but attractive: dimensions of new venture employer attractiveness and the moderating role of applicants’ entrepreneurial be-haviours. *J. Bus. Ventur.* 32 588–610. 10.1016/j.jbusvent.2017.05.001

[B45] Nayak S. Suhan. (2017). Antecedents to employer branding: a strategic focus on the information technology (IT) sector in India. *Pol. J. Manag. Stud.* 15 143–151. 10.17512/pjms.2017.15.2.13

[B46] O’Neill J. W. Harrison M. M. Cleveland J. Almeida D. Stawski R. Crouter A. C. (2009). Work-family climate, organisation commitment, and turnover: multilevel contagion effects of leaders. *J. Vocat. Behav.* 74 18–29. 10.1016/j.jvb.2008.10.004 19412351 PMC2675786

[B47] Page M. J. McKenzie J. E. Bossuyt P. M. Boutron I. Chou R. Shamseer L. (2021). The PRISMA 2020 statement: an updated guideline for reporting systematic reviews. *BMJ* 18:e1003583. 10.1136/bmj.n71 33780438 PMC8007028

[B48] Poulis A. Wisker Z. (2016). Modeling employee-based brand equity (EBBE) and per-ceived environmental uncertainty (PEU) on a firm’s performance. *J. Prod. Brand Manag.* 25 490–503. 10.1108/jpbm-04-2015-0852

[B49] Reis G. G. Braga B. M. Trullen J. (2017). Workplace authenticity as an attribute of employer attractiveness. *Pers. Rev.* 46 1962–1976. 10.1108/pr-07-2016-0156

[B50] Robertson J. Ferguson S. L. Eriksson T. Näppä A. (2019). The brand personality dimensions of business-to-business firms: a content analysis of employer reviews on social media. *J. Bus. Bus. Mark.* 26 109–124. 10.1080/1051712x.2019.1603354

[B51] Roongrerngsuke S. Liefooghe A. (2013). Attracting gold-collar workers: comparing organisational attractiveness and work-related values across generations in China. India and Thailand. *Asia Pac. Bus. Rev.* 19 337–355. 10.1080/13602381.2012.747784

[B52] Sahu S. Pathardikar A. Kumar A. (2018). Transformational leadership and turnover: mediating effects of employee engagement, employer branding, and psychological attachment. *Leadersh. Organ. Dev. J.* 39 82–99. 10.1108/lodj-12-2014-0243

[B53] Schlager T. Bodderas M. Maas P. Cachelin J.-L. (2011). The influence of the employer brand on employee attitudes relevant for service branding: an empirical investiga-tion. *J. Serv. Mark.* 25 497–508. 10.1108/08876041111173624

[B54] Sengupta A. Bamel U. Singh P. (2015). Value proposition framework: implications for employer branding. *Decision* 42 307–323. 10.1007/s40622-015-0097-x

[B55] Slatten T. Lien G. Svenkerud P. J. (2019). The role of organisational attractiveness in an internal market-oriented culture (IMOC): a study of hospital frontline employees. *BMC Health Serv. Res.* 19:307. 10.1186/s12913-019-4144-8 31088463 PMC6518731

[B56] Tanwar K. Prasad A. (2016). The effect of employer brand dimensions on job satisfac-tion: gender as a moderator. *Manag. Decision* 54 854–886. 10.1108/md-08-2015-0343

[B57] Tanwar K. Prasad A. (2017). Employer brand scale development and validation: a se-cond-order factor approach. *Pers. Rev.* 46 389–409. 10.1108/pr-03-2015-0065

[B58] Theurer C. P. Tumasjan A. Welpe, Isabell M. Lievens F. (2018). Employer branding: a brand equity-based literature review and research agenda. *Int. J. Manag. Rev.* 20 155–179. 10.1111/ijmr.12121

[B59] Trybou J. Gemmel P. Van Vaerenbergh Y. Annemans L. (2014). Hospital-physician relations: the relative importance of economic, relational and professional attributes to organisational attractiveness. *BMC Health Serv. Res.* 14:232. 10.1186/1472-6963-14-232 24884491 PMC4055796

[B60] Tumasjan A. Kunze F. Bruch H. Welpe I. M. (2019). Linking employer branding orientation and firm performance: testing a dual mediation route of recruitment efficiency and positive affective climate. *Hum. Res. Manag.* 59 83–99. 10.1002/hrm.21980

[B61] Turban D. B. Greening D. W. (1997). Corporate social performance and organisational attractiveness to prospective employee. *Acad. Manag. J.* 40 658–672. 10.5465/257057

[B62] Uen J. F. Ahlstrom D. Chen S. Y. Liu J. (2015). Employer brand management, organisational prestige and employees’ word-of-mouth referrals in Taiwan. *Asia Pac. J. Hum. Res.* 53 104–123. 10.1111/1744-7941.12024

[B63] Ul Hadi N. Ahmed S. (2018). Role of employer branding dimensions on employee retention: evidence from educational sector. *Adm. Sci.* 8:44. 10.3390/admsci8030044

[B64] Urbancova H. Hudakova M. (2017). Benefits of employer brand and the supporting trends. *Econ. Sociol.* 10 41–50. 10.14254/2071-789x.2017/10-4/4

[B65] Van Hoye G. (2013). Recruiting through employee referrals: an examination of employees’. Motives. *Hum. Perform.* 26 451–464. 10.1080/08959285.2013.836199

[B66] Van Hoye G. Lievens F. (2007). Social influences on organisational attractiveness: investigating if and when word of mouth matters. *J. Appl. Soc. Psychol.* 37 2024–2047. 10.1111/j.1559-1816.2007.00249.x

[B67] Van Hoye G. Lievens F. (2009). Tapping the grapevine: a closer look at word-of-mouth as a recruitment source. *J. Appl. Psychol.* 94 341–352. 19271794 10.1037/a0014066

[B68] Van Hoye G. Turban D. B. (2015). Applicant-employee fit in personality: testing predictions from similarity-attraction theory and trait activation theory. *Int. J. Sel. Assess.* 23 210–223. 10.1111/ijsa.12109

[B69] Van Vianen A. E. M. (2000). Person-organisation fit: the match between newcomers’ and recruiters’ preferences for organisational cultures. *Pers. Psychol.* 53 113–149. 10.1111/j.1744-6570.2000.tb00196.x

[B70] Vnouckova L. Urbancova H. Smolova H. (2018). Building employer image thanks to talent programmes in Czech organisations. *Inzinerine Ekon. Eng. Econ.* 29 319–331.

